# Differential self-reported COVID-19 impacts among U.S. secondary teachers by race/ethnicity

**DOI:** 10.3389/feduc.2022.931234

**Published:** 2022-07-22

**Authors:** Erin Josephine McCauley, Alexandra Cooperstock

**Affiliations:** 1 Department of Social and Behavioral Sciences, Philip R. Lee Institute for Health Policy Studies (Affiliated Faculty), University of California, San Francisco, San Francisco, CA, United States; 2 Department of Sociology, Cornell University, Ithaca, NY, United States

**Keywords:** COVID-19, education, teachers, teacher workforce, psycho-social

## Abstract

The COVID-19 pandemic created drastic changes for public education in the United States, including the role and responsibilities of educators. This study explores the self-reported psycho-social implications of COVID-19 among U.S. secondary teachers who are white, Black, Indigenous, and people of color. Using a national survey (*n* = 1,478) fielded between October 2020 and March 2021, we capture teachers’ self-reported level of concern, life change, impact on thinking, and impact on teaching ability due to the COVID-19 pandemic. While teachers who are Black, Indigenous, and people of color report higher levels of concern and daily life change stemming from COVID-19, they report lower impacts on their teaching ability relative to their white peers. These findings are consistent with racial/ethnic disparities in COVID-19 case rates and mortality and highlight the resiliency of the U.S. secondary teachers who are Black, Indigenous, and people of color.

## Introduction

Since March of 2020, the school systems in the United States (U.S.) have experienced substantial change and instability due to the onset of the SARS-CoV-2 virus, commonly referred to as the COVID-19 pandemic. Environments, routines, and instructional approaches have shifted as teachers have needed to navigate unfamiliar technology and adapt to combinations of socially distanced in-person classrooms, hybrid teaching, or virtual learning—all while managing personal and familial health concerns (Turchi et al., 2020). Most teachers in the U.S. were working full or part-time remote, substantially impacting their day-to-day activities (Kraft and Simon, 2020). The present study interrogates the psycho-social impacts of the COVID-19 pandemic among U.S. secondary teachers, exploring how the pandemic has shaped teachers’ daily lives, teaching ability, cognitive ability, and level of concern.

### Ethnoracial disparities in COVID-19

As the global health pandemic began to unfold, ethnoracial disparities in rates of COVID-19 cases and deaths became central in the U.S. national conversation (Chowkwanyun and Reed, 2020). In the first year and a half of the pandemic, Black individuals experienced the highest burden of COVID-19 in the U.S., with a prevalence ratio of 1.8, a hospitalization ratio of 1.9, and a mortality ratio of 1.7, compared to whites (Mude et al., 2021). Hispanics and other racial/ethnic groups also experienced elevated ratios relative to whites, especially for hospitalizations (Hispanic = 1.3 and other = 1.1) (Mude et al., 2021). Additionally, Black and Hispanic Americans were more likely to report knowing someone who had died of COVID-19 (39% and 31%, respectively) compared to white Americans (18%) (Ipsos, 2020).

Given the disproportionate impact of COVID-19 on people of color, it is reasonable to assume that non-white teachers are facing more challenges than their white counterparts, inside and outside of their classroom walls. Teaching during a global health pandemic is problematic, but it may not be a burden shared equally or in the same way among all U.S. educators. Indeed, it is still an open question as to whether the COVID-19 context and associated changes have had differential psycho-social consequences for white and non-white teachers.

### Importance of psycho-social impacts

The psycho-social impacts of COVID-19 for teachers are important for a few reasons. First, these impacts may contribute to losses in the teacher workforce. Before the pandemic, more than 44 percent of new teachers left the classroom within their first 5 years (Ingersoll et al., 2021). A handful of factors translate into losses for the U.S. teaching workforce—high levels of stress and emotional labor paired with low salaries and autonomy can yield feelings of disempowerment, devaluation, and dissatisfaction. Challenges which have arguably only been compounded since the start of the COVID-19 pandemic. Indeed, one out of four teachers in the U.S. considered leaving their jobs during the 2020–2021 school year, a marked increase from before the onset of the COVID-19 pandemic (Steiner and Woo, 2021).

Beyond the immediate staffing impact, the acceleration of teacher workforce losses could also hurt student performance. Research indicates that teacher turnover negatively impacts students’ academic success (Hanushek et al., 2016). The negative effect of teacher turnover is particularly strong in lower-performing schools and among Black students (Ronfeldt et al., 2013), suggesting that an acceleration of teacher turnover could also contribute to–and exacerbate–existing disparities in education.

Furthermore, given the ethnoracial differences in COVID-19 exposure and mortality in the general population (Mude et al., 2021), it is possible that teachers who are Black, Indigenous, and people of color are more impacted by the COVID-19 pandemic, and therefore may be more likely to leave the profession. There is already a substantial diversity gap in the U.S., with 40% of school-aged students being people of color compared to only 17% of their teachers (Boser, 2014). This diversity gap exists in every state and has increased over time (Boser, 2014). Teacher diversity is an important policy tool for closing achievement gaps and realizing educational equity (Gershenson et al., 2021). Understanding the ethnoracial differences in the psycho-social impacts of COVID-19 on teachers could be important in preserving the diversity of the teacher workforce.

Additionally, the psycho-social impacts of COVID-19 may affect teacher effectiveness. It has long been known that teacher stress effects performance (Blase, 1986). There have been documented increases in teacher stress rates over time (Markow and Scheer, 2003) and teaching is considered one of the most high stress professions (Gallup, 2014). Studies in multiple European countries found that teachers experienced further increased levels of stress at the start of the COVID-19 pandemic (Kim and Asbury, 2020; Pöysä et al., 2021). In the U.S., the anxiety wrought by COVID-19 is contributing to teacher burnout (Pressley, 2021) and the pandemic even changed teachers’ occupational identities and conceptualizations of teacherhood (Jones and Kessler, 2020). Therefore, the psycho-social impacts of COVID-19 may translate to decreased teacher effectiveness.

### The present study

Psycho-social impacts of COVID-19 are likely to contribute to teacher retention issues and related staffing shortages. Importantly, differential psycho-social impacts for teachers who are Black, Indigenous, and people of color, compared to their white counterparts, may shape the diversity of the teacher workforce through differential departures from the profession, adding additional urgency to this question. In the present study, we aim to: (1) Examine the psycho-social consequences of COVID-19 among U.S. secondary teachers; and (2) Explore differential impacts among teachers who are white, Black, Indigenous, and people of color. We expect that the global health pandemic has created salient psycho-social consequences for U.S. secondary teachers and has impacted the lives of teachers who are Black, Indigenous, and people of color more negatively than their white counterparts, given disproportionate rates of COVID-19 infection and mortality. Understanding how COVID-19 has impacted teachers’ lives, teaching ability, thinking ability, and their level of concern over the pandemic will provide vital insights into the broader costs of the pandemic for the teacher workforce, teacher diversity, and student learning.

## Methods

### Data

The data used in this study were collected by the research team as a part of a survey with secondary teachers in the United States. This survey was fielded between October of 2020 and March of 2021. The survey was conducted using Qualtrics and the project received ethical committee approval by the BLINDED Institutional Review Board. Participants were recruited from three school districts in each state in the U.S.^1^ (*n_districts_* = 150). Districts were identified for recruitment based on student population and achieving a mix of rurality in each state, as well as the public availability of teacher subjects and email addresses. Recruitment districts are kept anonymous to protect participant confidentiality. Email addresses were collated from publicly available information found on school district websites. Participants were invited to complete a survey about how teachers assess students and their work through an email invitation. Participation was anonymous, and only non-identifiable demographic information was collected. All participants completed informed consent prior to completing the survey. Participants received a $10 Amazon gift card as an incentive.

Teachers in public secondary schools listed as teaching core subjects were considered eligible to recruit. Core subjects are English/Language Arts, Social Studies/Government/History, Science, and Mathematics. Participants with missing racial/ethnic data (*n* = 102) are excluded from our analyses below. Descriptive statistics for our final analytic sample are reported in Table 1 (*n* = 1,478). The sample is, on average, 40 years old (*sd* = 12, range = 20–80), has been teaching for nearly 13 years (*sd* = 9, range = 1–47), and is a third male. The most common subjects represented are math / science (41%) and English (30%), and the most common regions are the Southeast (23%) and West Coast (20%). While these data are not nationally representative–the sample is not a random sample–they do provide a national perspective.

### Measures

This study explores psycho-social impacts of COVID-19. Survey participants answered questions to self-identify how COVID-19 has impacted their personal and professional lives, as seen in Appendix Table A1. The four questions include: (1) How concerned they are about the pandemic; (2) How much the pandemic changed their lives; (3) How much the pandemic impacted their ability to think clearly; and (4) How much the pandemic impacted their ability to teach. Responses ranged from one to five, with one representing the lowest impact (not at all) and five the highest (very).

Basic information on participants was collected in the survey, including gender, age, years teaching, primary teaching subject (English, history/government, math/science, special education, or electives), and region (West Coast, Rocky Mountains, Southwest, Plains, Great Lakes, Southeast, Mideast, or New England). Data were also collected on participant race and ethnicity (non-Hispanic White, non-Hispanic Black, Hispanic, non-Hispanic Asian, and other race). Using this data, participants were classified into two categories: Either a white category or a category consisting of Black, Indigenous, and people of color.

We examine the mean scores for each question for the full sample, as well as for Black, Indigenous, and people of color and whites separately. We then conduct independent sample *t*-tests to examine if these group averages are significantly different from one another.

## Results

Table 2 presents the mean values for each COVID-19 impact question for the full sample, and Black, Indigenous, and people of color and whites separately, in addition to the results from the independent sample *t*-tests. The overall COVID-19 impact was self-reported to be, on average, between “somewhat” and “quite” strong. However, this mean of 3.55 for the full sample of secondary U.S. teachers obscures some variability by question and race/ethnicity.

The lowest impact measure assesses the perceived relationship between COVID-19 and the educators’ ability to think clearly. On average, the full sample of secondary teachers self-reported that their ability to think clearly was only “slightly” or “somewhat” influenced by the global health pandemic (a value of 2.37). The mean values for teachers who are Black, Indigenous, and people of color are not statistically significantly different from white teachers.

Despite the self-reports of only a modest impact of COVID-19 on their ability to think clearly, the secondary teachers surveyed indicated a stronger relationship between the global health pandemic and their personal lives in other realms. Respondents indicated that, on average, their daily lives had changed “quite” a lot or “very” much as a result of COVID-19 and that they were “quite” concerned overall (mean values of 4.35 and 3.83, respectively). These feelings of tumultuousness and unease carried over into their professional lives as well. Respondents indicated that COVID-19 “somewhat” or “quite” impacted their teaching ability, on average (mean value of 3.65).

While teachers who are Black, Indigenous, and people of color and teachers who are white both reported concern regarding COVID-19 and its effects on their personal and professional lives, the results of our independent sample *t*-tests indicate that the level of concern between these racial/ethnic groups were significantly different. Teachers who are Black, Indigenous, and people of color reported, on average, higher levels of concern (4.01 compared to 3.79; *p* < 0.01) and more awareness that their lives have been altered (4.45 compared to 4.33; *p* < 0.05) than their white counterparts. However, despite a greater salience of the global health pandemic by teachers who are Black, Indigenous, and people of color, they reported that COVID-19 had a lower impact on their ability to teach than white teachers (3.51 compared to 3.69; *p* = 0.03).

## Discussion

Our first aim is to assess the self-reported psycho-social impacts of COVID-19 among U.S. secondary teachers. Not surprisingly, we find that teachers report high levels of impact across outcomes (mean value of 3.55). This is consistent with existing research documenting changes to U.S. education during this time period (Turchi et al., 2020) and the very real threat of the COVID-19 pandemic (Jin et al., 2021). Teachers reported higher impact on life change (4.35), concern (3.83), and teaching ability (3.65), relative to thinking ability (2.37). This suggests lower cognitive impacts than other psycho-social impacts. Teacher burnout and stress are large contributors to losses in the teacher workforce (Ingersoll, 2002). Taken together, the psycho-social impacts of COVID-19 may contribute to teacher workforce losses and future research should explore this possibility.

In terms of our second aim, the findings provide support for our hypothesis; secondary teachers who are Black, Indigenous, and people of color indicated that COVID-19 had a greater impact than their white counterparts for some outcomes. Teachers who are Black, Indigenous, and people of color reported a higher impact of COVID-19 on their daily lives and higher levels of concern about COVID-19. These results are congruent with research findings that Black, Indigenous, and people of color were more likely to wear masks and comply with social distancing (Gette et al., 2021). Furthermore, given the disproportionate infection rate and mortality rate, the higher level of concern about COVID-19 among teachers who are Black, Indigenous, and people of color is justified (Chowkwanyun and Reed, 2020; Jin et al., 2021).

It is important to note though that despite these differences, white teachers actually reported that COVID-19 has had a stronger impact on their ability to teach than teachers who are Black, Indigenous, and people of color. While we did not expect this result, it is not necessarily surprising given the plethora of other ways teachers who are Black, Indigenous, and people of color are forced to demonstrate resilience and perseverance in U.S. majority white schooling institutions. This is especially of note given the importance of teacher resilience for retention and effectiveness (Gu and Day, 2007). Future research should examine if the differential psycho-social impacts of COVID-19 on white teachers and teachers who are Black, Indigenous, and people of color identified in this study translates to differential retention, or if the resilience of educators who are Black, Indigenous, and people of color may mitigate this risk.

Extending upon the body of research on the resilience of urban educators (e.g., Stanford, 2001; Brunetti, 2006), future research should explore how teachers fostered resilience during the global health pandemic, especially non-white teachers who face disproportionate impacts of COVID-19, in addition to the obligations felt by all teachers. These results help us to reconceptualize the narrative about the burdens the teaching workforce is currently facing and reframes the conversation to focus on teacher resilience, especially the resilience of educators who are Black, Indigenous, and people of color.

### Limitations

While the sampling design used in this survey captures a national perspective, it is not nationally representative and should not be viewed in this light. Participants were selected for recruitment based on the size of the district, the general urbanicity or rurality of the district to achieve a balance in each state, and the public availability of emails on school websites. This is a convenience sample, not a random sample. The recruitment strategy relied on teachers receiving, opening, and agreeing to participate in a survey from an invitation from an email address outside of the school district. The risks of cyber threats in secondary schools increased during the COVID-19 pandemic, which likely impacted both teachers’ willingness to participate and their assessment of whether the invitation was “spam” or not. Additionally, many websites were not kept up to date during the pandemic due to enhanced stress on school resources, which may have translated into invitations being sent out to non-active email addresses. Due to the lack of information about which teachers actually received the invitation, a traditional response rate is impossible to calculate. Therefore, the results here should not be generalized to the entire population.

It is important to note, however, that the characteristics of teachers in this sample are similar to national characteristics providing credence to the national perspective of these results. According to the National Center for Education Statistics, 80% of U.S. secondary high school teachers are non-Hispanic white compared to 79% in this sample; 36% of U.S. secondary high school teachers are male compared to 32% in this sample; U.S. secondary high school teachers have 14 years of teaching experience, on average, compared to 11 in this sample; and the average age of a U.S. secondary high school teacher is 42 years old, compared to 40 years old in this sample. These similarities provide some assurances that this sample is similar to the characteristics of teachers nationally.

Despite our sampling limitations, this study delivers detailed information about the impact of COVID-19 on U.S. secondary teachers. It also provides important context in which to view those impacts and hypothesize about the downstream consequences for the diversity of the teaching workforce. Future research should build on these findings, probing and interrogating potential explanations for the differences between educators who are Black, Indigenous, and people of color and educators who are white captured in this study. Moreover, research should disaggregate the Black, Indigenous, and people of color category to look at different racial/ethnic groups. Unfortunately, we lacked the sample size to conduct these important comparisons in this study. Last, future research should build on our hypothesis that teachers who are Black, Indigenous, and people of color were more impacted in their personal lives by the COVID-19 pandemic, but less so in their professional lives. Research could more directly measure teaching quality and examine differential departures from the secondary teacher workforce by racial and ethnic groups.

## Conclusion

School districts and administrators should be especially responsive to what their teachers are currently experiencing. This includes a deeper understanding of differential working conditions in addition to differential perceptions of them, and how each of these are tied to wellbeing, effectiveness, and retention. Our study speaks to this, and also sheds light on how a diversity of interventions may be needed to address differential psycho-social impacts. This is essential for two reasons. First, even if teachers remain in the profession, declining job satisfaction could affect teacher quality and hinder students’ academic progress. Second, despite the recent diversification of U.S. teachers, the teaching workforce still does not reflect the student population overall (Ingersoll et al., 2021). The question of varying levels of retention by race/ethnicity in the U.S. teaching workforce in a post-COVID-19 world is of consequence as diversity has been found to be one core component of teacher quality (Gershenson et al., 2021). Access to same-race teachers improves test scores, increases chances of high school graduation and referrals to gifted and talented programs, and lowers absenteeism. Diversifying the teaching workforce yields positive effects for white students and teachers as well. Teacher diversity is an important policy lever which is vital in efforts to close the race-based achievement gap in the U.S. and move schools closer to equity goals. While U.S. secondary teachers who are Black, Indigenous, and people of color and U.S. secondary teachers who are white are experiencing substantial and differential psycho-social impacts of COVID-19, the downstream consequences for teacher retention and student performance remain unknown. Policy and practice efforts should focus on fostering, and learning from, the resiliency of teachers who are Black, Indigenous, and people of color to promote teacher retention.

## Figures and Tables

**TABLE 1:** Descriptive statistics.

	Full sample		White		BIPOC	
	Mean/Proportion	*SD*	Mean/Proportion	*SD*	Mean/Proportion	*SD*
Male	0.33	–	0.34	–	0.30	–
Age	39.65	11.72	40.08	11.66	37.75	11.99
Years teaching	12.80	9.47	13.17	9.49	10.98	9.28
Subject						
English	0.30	–	0.30	–	0.30	–
History/government	0.17	–	0.18	–	0.12	–
Math/science	0.41	–	0.42	–	0.42	–
Special education	0.04	–	0.04	–	0.06	–
Electives	0.08	–	0.07	–	0.10	–
Region						
West coast	0.20	–	0.16	–	0.35	–
Rocky mountains	0.13	–	0.14	–	0.07	–
Southwest	0.06	–	0.06	–	0.07	–
Plains	0.12	–	0.13	–	0.08	–
Great lakes	0.09	–	0.11	–	0.05	–
Southeast	0.23	–	0.23	–	0.27	–
Mideast	0.10	–	0.10	–	0.02	–
New England	0.07	–	0.07	–	0.09	–
	*N* = 1,478		*N* = 1,221		*N* = 257	

BIPOC stands for Black, Indigenous, and people of color. The Black, Indigenous, and people of color category is comprised of 28% Black, 28% Hispanic, 24% Asian, and 20% other.

**TABLE 2:** Means tests.

	Full sample		White		BIPOC		White—-BIPOC *T*-test	
	Mean	*SD*	Mean	*SD*	Mean	*SD*	Difference	*P*-value
Concerned	3.83	1.21	3.79	1.22	4.01	1.15	−0.22**	0.01
Changed life	4.35	0.88	4.33	0.89	4.45	0.84	−0.12*	0.05
Think clearly	2.37	1.27	2.40	1.27	2.30	1.26	0.10	0.37
Teaching ability	3.65	1.22	3.69	1.20	3.51	1.32	0.18*	0.03
*Average impact*	3.55	0.81	3.55	0.81	3.57	0.81	−0.02	0.72
	*N* = 1,478		*N* = 1,221		*N* = 257			

BIPOC stands for Black, Indigenous, and people of color.

* p < 0.05,

** p < 0.01.

## Data Availability

The raw data supporting the conclusions of this article will be made available by the authors, without undue reservation.

## Footnotes

1 In Hawaii, which has one state-wide school district, we recruited from three complex districts.

## Appendix

**APPENDIX TABLE A1:** COVID-19 Survey questions.

	Not at all (1)	Slightly (2)	Somewhat (3)	Quite (4)	Very (5)	N/A
1) How concerned are you about COVID-19?	◦	◦	◦	◦	◦	◦
2) How much has COVID-19 changed your daily life?	◦	◦	◦	◦	◦	◦
3) How much has the threat of COVID-19 decreased your ability to think clearly?	◦	◦	◦	◦	◦	◦
4) How much has COVID-19 affected your ability to teach?	◦	◦	◦	◦	◦	◦

I know this survey comes at an unusual time, with the country facing the global pandemic of COVID-19.

This pandemic has affected everyone differently and the last few questions are about how COVID-19 has affected you.
